# Association between serum cystatin C level and post‐stroke cognitive impairment in patients with acute mild ischemic stroke

**DOI:** 10.1002/brb3.2519

**Published:** 2022-02-11

**Authors:** Xu Yan, Huan Chen, Xiu‐Li Shang

**Affiliations:** ^1^ Department of Neurology The First Affiliated Hospital of China Medical University and The First people’s Hospital of Shenyang. Shenyang China; ^2^ Department of Neurology The First people’s Hospital of Shenyang Shenyang China; ^3^ Department of Neurology The Fourth Affiliated Hospital of China Medical University Shenyang China

**Keywords:** cognitive impairment, cystatin C, mild ischemic stroke, Montreal Cognitive Assessment

## Abstract

**Background:**

Mild ischemic stroke (MIS) has been proved to be closely related to post‐stroke cognitive impairment (PSCI). However, there are relatively few studies on the risk factors of MIS. We aimed to evaluate the relationship between serum cystatin C (CysC) level and cognitive function in patients with acute MIS.

**Methods:**

Four hundred consecutive patients with acute MIS were screened and 281 patients were eligible for this study. The serum CysC levels were detected within 24 h after admission. Cognitive function was assessed by Montreal Cognitive Assessment (MoCA) at 3 months after acute MIS. Logistic regression was used to identify the predictors of PSCI, and the receiver operating characteristic (ROC) curve was applied to explore the optimal cut‐off value.

**Results:**

One hundred sixty‐four (58.4%) patients were diagnosed with PSCI at 3 months follow‐up. The serum CysC levels in patients with PSCI were significantly higher than patients without PSCI (*p* < .001). The binary logistic regression analysis showed that higher serum CysC level was an independent predictor for PSCI at 3 months (odds ratio [OR], 5.745; 95% confidence interval, [CI], 1.089–30.311; *p* = 0.039). The ROC curve showed that area under the curve (AUC) was 0.723, and at a 0.945 mg/l CysC cut‐off point, the sensitivity and specificity for PSCI at 3 months were 79.9% and 58.1%, respectively.

**Conclusion:**

Our findings suggested that the serum CysC levels were increased after acute MIS, and higher serum CysC levels at baseline might be an independent risk factor for PSCI in patients with acute MIS, but further research are warranted.

## Graphical abstract

The serum CysC level was increased after acute MIS, and elevated CysC level was closely related to the occurrence of PSCI in MIS patients. It was also found to have high sensitivity and specificity through ROC curve.

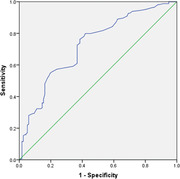

## 1 INTRODUCTION

Stroke is one of the diseases with the highest morbidity in the world. Although the total morbidity of stroke has decreased in recent years, the absolute number of strokes is gradually increasing. It is the main cause of disability or death of human beings and poses a serious threat to human health (J. Kim et al., 2020; Lindsay et al., 2019; Wafa et al., 2020); therefore, stroke prevention and control are urgently needed (Chao et al., 2021). Post‐stroke cognitive impairment (PSCI) is a common symptom of stroke, and it affects 20%–80% of patients, depending on countries, ethnicities, and diagnostic criteria (Sun et al., 2014). At the same time, PSCI is also an independent predictor of recurrent ischemic stroke, which affects the quality of life and prognosis of stroke survivors (Kwon et al., 2020).

Mild ischemic stroke (MIS) is characterized by mild symptoms and few neurological deficits. In China, one out of every three patients with acute ischemic cerebrovascular disease are MIS patients (Chen et al., 2017). Different studies have different views on the definition of MIS, and there is no unified view up to now, among which, most studies define stroke with NIHSS score <5 as MIS (Roberts et al., 2020). Previous studies have shown that PSCI has a higher prevalence in patients with MIS (J. Li et al., 2019). However, few studies have been conducted on the risk factors for PSCI of MIS patients. PSCI has brought an extremely heavy burden to an increasingly aging society, and more attention should be paid to the research on the risk factors of PSCI.

Cystatin C (CysC), a member of the endogenous cysteine protease inhibitor family, is produced at a steady charge in all nucleated cells (T. J. Kim et al., 2017). CysC has been proved to be a better marker than creatinine, and it is considered as a potential substitute to serum creatinine for estimating glomerular filtration rate (GFR) (Inker et al., 2012). At present, the relationship between serum CysC level and PSCI is not clear. Some scholars (Guo et al., 2020) believe that CysC is a protective factor of nervous system diseases and a new target for the treatment of stroke. However, others suggest that high serum CysC level can increase the risk of vascular dementia after acute cerebral infarction (Zeng et al., 2019). Further research is needed to clarify the relationship between the two. At the same time, the relationship between the serum CysC level and PSCI in patients with MIS has not been reported. Therefore, we performed this prospectively study to evaluate the association between CysC and PSCI at 3 months after MIS.

## 2 METHODS

### 2.1 Study design and patients

We prospectively collected the demographic and clinical data of patients with acute MIS who were admitted to the First Affiliated Hospital of China Medical University from September 2019 to August 2020. The inclusion criteria were as follows: (1) age ≥18 years old, (2) those who were first diagnosed as acute ischemic stroke by magnetic resonance imaging within 1 week after stroke, and (3) NIHSS score <5. The exclusion criteria were as follows: (1) patients with cerebral hemorrhage, (2) patients with a record of any central nervous system diseases, and (3) patients who have difficulty in hearing, seeing, and speaking and refuse to cooperate with clinical examination. We also excluded the patients who were treated with endovascular therapy or intravenous thrombolysis during hospitalization. This study was approved by the Ethics Committee of the First Affiliated Hospital of China Medical University (number: 2021–457) and was conducted in accordance with the Declaration of Helsinki. Written informed consent was provided by all participants or their legal representatives.

Baseline data on demographic characteristics and clinical features were collected at the time of admission. Stroke severity was assessed by the National Institutes of Health Stroke Scale (NIHSS). Blood samples were collected after fasting for at last 8 h within 24 h of admission. Biochemical parameters were measured on an automated hematology analyzer. The serum CysC levels were measured by a high sensitivity latex‐enhanced immunoturbidimetric method with an automatic biochemical analyzer by experienced laboratory physicians. The reference value of serum CysC ranged from 0.53 to 0.95 mg/L. Intra‐ and interassay coefficients of variation were less than 3.9% and 4.8%, respectively.

### 2.2 Assessment of outcomes

The main outcome was cognitive impairment at 3 months after acute MIS. Cognitive function was assessed by Montreal Cognitive Assessment (MoCA). The assessment process was conducted by trained neurologists who were blinded to the laboratory results and clinical data. Thirty points were the total score of this scale, and the lower the score, the more severe the cognitive impairment (Mijajlović et al., 2017). On the basis of criteria, cognitive function was classified as follows: no cognitive impairment was defined by scores from 27 to 30, and post‐stroke cognitive impairment‐no dementia (PSCI‐ND) was defined by scores from 23 to 26 and post‐stroke dementia (PSD) was defined by scores from 0 to 22 (Mijajlović et al., 2017; Pendlebury et al., 2010). In this analysis, a score of ≤26 on the MoCA indicated cognitive impairment.

### 2.3 Statistical analysis

All patients were categorized into four subgroups according to the quartile of serum CysC levels, and baseline data were compared among the four groups. Continuous variables were expressed as means with SD or median with interquartile range (IQR). Differences between groups were tested using the independent‐samples *t*‐test or analysis of variance (ANOVA). The correlation between the two variables was analyzed by Pearson correlation analysis. Categorical variables were described by frequencies with percentages, and the *χ* ^2^ test was used to examine differences. The effect of CysC on the presence of PSCI was evaluated by binary logistic regression analysis including factors with statistically significant differences (*p* < .05) in the univariate analysis between groups. Results were shown as adjusted odds ratio (OR) (95% confidence interval, CI). The receiver operating characteristic (ROC) curve was applied to explore the optimal cut‐off value of serum CysC levels for predicting PSCI. For all the analyses, *p* < .05 was regarded statistically significant. Statistical analyses were performed using the SPSS program (Version 21.0, IBM Statistics).

## 3 RESULTS

**FIGURE 1:**
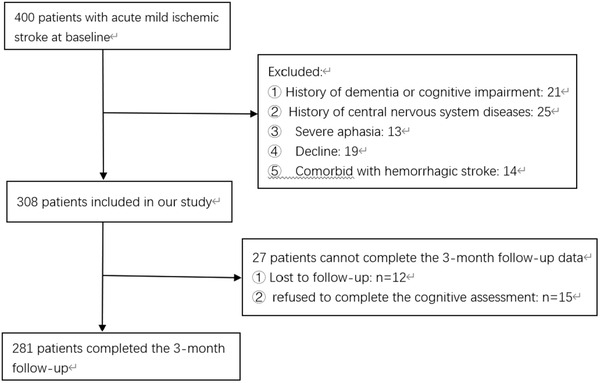
Study recruitment profile

During September 2019 to August 2020, 400 consecutive patients with acute MIS were screened and 308 patients were included in this study. Twenty‐seven patients were lost to follow up or refused to complete the scale at 3 months. Thus, 281 patients were eligible for this study (Figure 1). We presented the baseline characteristics of the 281 patients in Table 1. The quartile of serum CysC level is shown below: less than or equal to 0.9 mg/L (first quartile), 0.91–1.02 mg/L (second quartile), 1.03–1.21 mg/L (third quartile), and more than 1.21 mg/L (fourth quartile). There was a significant difference in sex (*p* = .010), hypertension (*p* = .018), age (*p*<.001), creatinine (*p*<.001), GFR (*p*<.001), homocysteine (*p*<.001), uric acid (*p*<.001), and urea nitrogen (*p*<.001) levels.

**TABLE 1:** Baseline characteristics of participants according to serum cystatin C (CysC) quartiles

	Cystatin C (mg/L)				
Variables*	≤0.90 (*n*=74)	0.91–1.02 (*n*=70)	1.03–1.21 (*n*=68)	>1.21 (*n*=69)	*p*
Sex (male)	40 (54.1)	51 (72.9)	49 (72.1)	54 (78.3)	.010
Age (year)	55.12 ± 12.24	60.94 ± 11.55	60.85 ± 13.12	65.09 ± 14.52	<.001
Years of education (year)	8.62 ± 1.69	7.9 ± 1.67	8.99 ± 8.71	7.23 ± 1.96	.110
Current smoking (*n*, %)	25 (33.8)	17 (24.3)	28 (41.2)	30 (43.5)	.078
Current drinking (*n*, %)	12 (16.2)	12 (17.1)	13 (19.1)	15 (21.7)	.840
Hypertension (*n*, %)	38 (51.4)	43 (61.4)	41 (60.3)	53 (76.8)	.018
Cardiovascular disease (*n*, %)	12 (16.2)	7 (10)	7 (10.3)	10 (14.5)	.610
Diabetes (*n*, %)	29 (39.2)	18 (25.7)	20 (29.4)	17 (24.6)	.208
Time from onset to randomization (hour)	32.28 ± 19.37	32.1 ± 20.93	29.13 ± 18.12	31.54 ± 18.49	.757
NIHSS at admission, median (IQR)	2 (1, 3)	1.5 (1, 3)	1 (1, 3)	2 (1, 3)	.191
Stroke etiology, *n* (%)					.556
Atherosclerosis	23 (31.1)	22 (31.4)	32 (47.1)	30 (43.5)	
Cardio embolism	2 (2.7)	3 (4.3)	1 (1.5)	2 (2.9)	
Small vessel occlusion	40 (54.1)	39 (55.7)	32 (47.1)	33 (47.8)	
Other undetermined etiology	1 (1.4)	1 (1.4)	1 (1.5)	0 (0)	
Undefined	8 (10.8)	5 (7.1)	2 (2.9)	4 (5.8)	
Laboratory variables uric acid, μmol/L	290.18 ± 83.58	311.93 ± 64.52	321.71 ± 71.45	367.25 ± 70.51	<.001
Urea nitrogen, mmol/L	4.7 ± 1.44	4.85 ± 1.19	6.15 ± 6.77	8.39 ± 8.58	<.001
GFR, ml/min per 1.73 m^2^	107.1 ± 11.31	100.58 ± 10.25	96.04 ± 13.48	70.88 ± 21.4	<.001
Creatinine, μmol/L	55.22 ± 12.32	60.84 ± 11.26	67.4 ± 11.2	100.39 ± 39.07	<.001
HDL‐cholesterol, mmol/L	1.13 ± 0.29	1.11 ± 0.28	1.1 ± 0.37	1.03 ± 0.27	.206
Triglyceride, mmol/L	1.59 ± 0.8	1.59 ± 1.19	1.67 ± 0.85	2.18 ± 3.73	.244
Total cholesterol, mmol/L	4.5 ± 1.21	4.56 ± 1.01	4.4 ± 1.09	4.55 ± 1.58	.864
LDL‐cholesterol, mmol/L	2.83 ± 1.04	2.9 ± 0.8	2.85 ± 0.82	2.75 ± 0.98	.816
Glycated hemoglobin, %	6.73 ± 1.95	6.7 ± 1.63	6.47 ± 1.57	6.49 ± 1.72	.736
Folic acid, nmol/L	19.29 ± 10.08	17.79 ± 10.2	16.14 ± 8.14	15.37 ± 8.58	.059
HCY, μmol/L	11.67 ± 6.06	13.59 ± 6.75	15.54 ± 9.12	19.82 ± 12.97	<.001
Hemoglobin, g/L	142.78 ± 16.86	142.15 ± 21.74	143.13 ± 23.78	139.64 ± 20.01	.751

Abbreviations: GFR, glomerular filtration rate; HCY, homocysteine; HDL, high‐density lipoprotein; IQR, interquartile range; NIHSS, National Institute of Health Stroke Scale; LDL, low‐density lipoprotein.

* Continuous variables are expressed as mean ± standard deviation or median (IQR). Categorical variables are expressed as frequency (%).

A total of 164 patients (58.4%) were diagnosed as PSCI at 3 ‐months follow‐up. We presented the baseline characteristics according to whether the patient has PSCI at 3 months in Table 2. As presented in Table 2, the level of serum CysC at 3 months was associated with PSCI by univariate analysis (*p*<.001). Patients in group with PSCI were older than those without PSCI (*p* = .004) and more likely to have diabetes (*p* = .034). There was also significant difference in uric acid (*p* = .018), GFR (*p*<.001), creatinine (*p* = .001), and homocysteine (*p* = .004). As shown in Table 3, after adjusting for age, diabetes, uric acid, GFR, creatinine, and homocysteine level, the logistic analysis showed that higher CysC level was an independent predictor for PSCI at 3 months (OR, 5.745; 95% CI, 1.089–30.311; *p* = .039).

**TABLE 2:** Characteristics of the patients with and without post‐stroke cognitive impairment (PSCI) at 3 months

Variables*	Without PSCI (*n* = 117)	With PSCI (*n* = 164)	*t*/*χ* ^2^	*p*
Sex (male)	77 (65.8)	117 (71.3)	0.977	.323
Age (year)	57.74 ± 12.2	62.31 ± 13.78	−2.877	.004
Years of education (year)	8.3 ± 1.59	8.11 ± 5.84	0.341	.733
Current smoking (*n*, %)	41 (35)	59 (36)	0.026	.872
Current drinking (*n*, %)	18 (15.4)	34 (20.7)	1.295	.255
Hypertension (*n*, %)	73 (62.4)	102 (62.2)	0.001	.973
Cardiovascular disease (*n*, %)	19 (16.2)	17 (10.4)	2.109	.146
Diabetes (*n*, %)	43 (36.8)	41 (25)	4.5	.034
Time from onset to randomization (hour)	31.79 ± 19.56	30.94 ± 19	0.364	.716
NIHSS at admission, median (IQR)	1.85 ± 1.24	1.99 ± 1.17	−0.959	.338
Stroke etiology, *n* (%)				
Atherosclerosis	38 (32.5)	69 (42.1)		
Cardio embolism	4 (3.4)	4 (2.4)		
Small vessel occlusion	66 (56.4)	78 (47.6)	3.03	.553
Other undetermined etiology	1 (0.9)	2 (1.2)		
Undefined	8 (6.8)	11 (6.7)		
Laboratory variables				
Uric acid, μmol/L	309.21 ± 79.07	331.38 ± 76.01	−2.369	.018
Urea nitrogen, mmol/L	6.43 ± 8.42	5.68 ± 2.04	0.953	.342
GFR, ml/min per 1.73 m^2^	100.27 ± 16.63	89.37 ± 21.11	4.838	<.001
Creatinine, μmol/L	64.26 ± 24.81	75.22 ± 29.15	−3.39	.001
HDL‐cholesterol, mmol/L	1.11 ± 0.31	1.08 ± 0.3	0.623	.534
Triglyceride, mmol/L	1.93 ± 2.94	1.63 ± 0.95	1.255	.210
Total cholesterol, mmol/L	4.57 ± 1.41	4.45 ± 1.09	0.762	.447
LDL‐cholesterol, mmol/L	2.83 ± 0.91	2.83 ± 0.92	−0.018	.986
Glycated hemoglobin, %	6.78 ± 1.82	6.48 ± 1.64	1.451	.148
Folic acid, nmol/L	17.67 ± 9.67	16.86 ± 9.2	0.715	.475
HCY, μmol/L	13.26 ± 8.09	16.4 ± 10.26	−2.864	.004
Hemoglobin, g/L	143.52 ± 18.67	140.81 ± 21.88	1.088	.277
CysC, mg/L	1 ± 0.38	1.2 ± 0.39	−4.375	<.001

Abbreviations: GFR, glomerular filtration rate; HCY, homocysteine; HDL, high‐density lipoprotein; IQR, interquartile range; NIHSS, National Institute of Health Stroke Scale; LDL, low‐density lipoprotein.

* Continuous variables are expressed as mean ± standard deviation or median (IQR). Categorical variables are expressed as frequency (%).

**TABLE 3:** Logistic regression analysis

Factor	*B*	SE	Wald	OR (95% CI)	*p*
Age	0.007	0.016	0.206	1.007 (0.977–1.039)	.65
Diabetes	0.478	0.286	2.808	1.614 (0.922–2.824)	.094
Uric acid	0.003	0.002	2.286	1.003 (0.999–1.007)	.131
GFR	−0.023	0.018	1.518	0.978 (0.943–1.013)	.218
Creatinine	−0.021	0.014	2.285	0.979 (0.953–1.006)	.131
HCY	0.021	0.017	1.466	1.021 (0.987–1.056)	.226
CysC	1.748	0.849	4.245	5.745 (1.089–30.311)	.039

Abbreviations: CI, confidence interval; CysC, cystatin C; GFR, glomerular filtration rate; HCY, homocysteine; OR, odds ratio.

**FIGURE 2:**
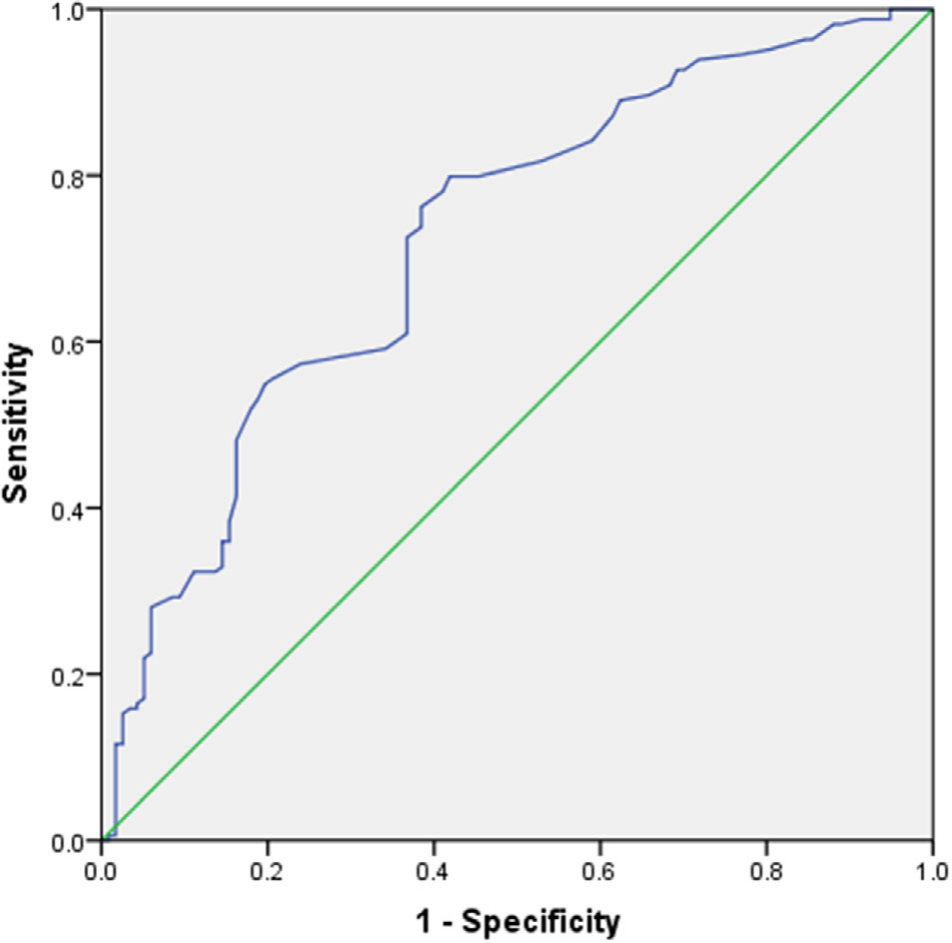
Receiver operating characteristic curve analysis of cystatin C (CysC) cut‐off point for the post‐stroke cognitive impairment (PSCI) at 3 months. Area under the curve (c‐statistic) = 0.723 (CysC)

The accuracy of CysC in the diagnosis of PSCI at 3 months was verified by ROC curve. The area under the curve (AUC) was 0.723. At a 0.945 mg/L CysC cut‐off point, the sensitivity and specificity for PSCI at 3 months were 79.9% and 58.1%, respectively (c‐statistic 0.723, 95% CI 0.663−0.783, *p* < .001, Figure 2).

## 4 DISCUSSION

In this prospective study, we explored the relationship between serum CysC and PSCI patients with MIS. Our data showed that serum CysC levels were increased after stroke, and the higher levels of serum CysC were associated with the presence of PSCI, and even after adjustment for potential confounding variables, CysC was independently related to the presence of PSCI. These results suggested that serum CysC could be a potential biomarker for PSCI in patients with MIS.

Previous studies (Fens et al., 2013; McHutchison et al., 2019) found that about 50% of patients with MIS showed a decline in their ability in daily work and to return to society, and about 40% of patients with MIS had cognitive dysfunction and communication difficulties. MIS is a type of stroke characterized by mild symptoms and short duration that rarely causes neurological deficits (Hamre et al., 2021), which are more likely to be overlooked. However, the higher incidence of PSCI in MIS patients would also bring serious harm to our society (Fens et al., 2013; McHutchison et al., 2019). As a result, the prevention of PSCI in MIS patients is particularly important. As far as we know, this is the first study exploring the relationship between CysC and cognitive impairment in patients with MIS.

In recent years, CysC, as a new biomarker of cardiovascular and cerebrovascular diseases, has attracted more and more attention (Hojs et al., 2014, 2018; Salgado et al., 2013). Some studies showed that CysC was increased in animal models of human nervous system diseases and neurodegenerative diseases (Hu et al., 2016; Xiong et al., 2018) and played an important role in the process of nervous system repair, which might be a target for the intervention of nervous system diseases (Amin et al., 2020; Ghidoni et al., 2010). In addition, prior research also reported that CysC was a significant determinant of endogenous neuroprotection, which might be a new drug candidate for the treatment of stroke by maintaining lysosomal membrane integrity (Fang et al., 2017). However, some research reported that CysC was a risk factor for stroke (Wang et al., 2019; Xiao et al., 2012). Compared with those without stroke, the level of serum CysC in patients with acute ischemic stroke was significantly higher, which can be used as a risk predictor of the acute ischemic stroke (Wang et al., 2019). In addition, it has also been suggested that higher CysC levels were associated with larger infarct size or blood volume and were considered as an independent predictor of infarct size and blood volume in acute ischemic stroke (Xiao et al., 2012). Whether CysC is a protective factor or a risk factor of stroke has not been consistently concluded up to now. Similarly, debates on the relationship of PSCI and serum CysC continue. A clinical study found that increased serum CysC level in patients with normal renal function was related to a lower risk of cognitive impairment at 3 months in a prospective observational study, suggesting that CysC was a protective factor for cognitive impairment (Guo et al., 2020). On the contrary, some studies indicated that serum CysC was a risk factor for cognitive impairment, and suggested that the serum CysC levels were closely associated with the development of Alzheimer's disease and mild cognitive impairment (Ghidoni et al., 2010; Sundelöf et al., 2010). Furthermore, the serum CysC levels were shown to be associated with long‐term dementia risk in older patients (Slinin et al., 2015). Another study suggested that high serum CysC level increased the risk of vascular dementia, and detection of CysC and CST3 gene polymorphism may contribute to the early diagnosis of vascular dementia (Zeng et al., 2019). However, few studies have been conducted on the relationship between CysC and cognitive impairment in patients with stroke, especially in patients with acute MIS. In our study, the patients we studied at 3 months follow‐up included PSCI‐ND and PSD patients, both of whom needed more attention and early prevention. We found that elevated CysC level was closely related to the occurrence of PSCI in MIS patients. As a clinical indicator, it was also found to have high sensitivity and specificity through ROC curve. Therefore, reducing the level of serum CysC may decrease the incidence of PSCI in MIS patients, which may also provide a new treatment for the prevention of PSCI, but further studies are needed to confirm it.

In accordance with the present results, the mechanisms by which serum CysC affects cognitive function after MIS remain unclear. We propose the following potential pathways. First, as our study has found, MIS can make an increase of serum CysC level. Previous studies showed that higher serum CysC level was closely related to dementia and cognitive dysfunction (Kono et al., 2017; Sundelöf et al., 2010; Yaffe et al., 2008). Second, one study showed that the serum CysC level in patients with artery stenosis was significantly higher than patients without artery stenosis, and there was a significant correlation between serum CysC level and cerebral artery stenosis (Xu et al., 2017). Similarly, higher CysC levels were found to be closely associated with the incidence of symptomatic common carotid artery stenosis and unstable plaques (Ren et al., 2020; Umemura et al., 2016). As is well known, carotid artery stenosis has been shown to be strongly associated with cognitive impairment in stroke and nonstroke patients (X. Li et al., 2017; Yue et al., 2016). In addition, CysC was involved in the pathogenic process of brain amyloidosis, which can lead to early cerebral hemorrhage (Levy et al., 2006). Previous study showed that the serum CysC level in patients with acute stroke complicated with intracranial microhemorrhage was significantly higher than patients without microhemorrhage and proved that CysC was an independent risk factor for intracranial microhemorrhage (Zhang et al., 2014). Intracranial microhemorrhage was closely related to cognitive dysfunction, causing damage to visual, spatial, and executive functions, as well as memory and abstract thinking (Yang et al., 2018). The discussion above may explain the possible mechanism of CysC and PSCI in patients with MIS. In our study, we confirm our hypothesis. Despite older age in PSCI group, CysC was independently associated with the presence of PSCI in patients with MIS after adjustment for potential confounding variables.

The different results of CysC and PSCI may be related to the different levels of CysC in cerebrospinal fluid (CSF) and serum. A previous study reported that the serum CysC levels were found five times higher in CSF than in serum (Sheikh & Nagai, 2019). However, to our knowledge, no studies have been conducted on the effect of stroke on CysC in CSF. Therefore, this will be our research direction in the future which shed light on the level of CysC in both CSF and serum in MIS patients.

There were some limitations in our study. First, serum CysC levels were measured only once, and it would be better to assess how CysC levels change over time after stroke. Second, the sample size was not large enough. Finally, we did not assess cognitive function before stroke, although patients with a record of any central nervous system diseases were excluded, cognitive function may be influenced by some potential confounding factors.

## 5 CONCLUSION

In summary, the current study revealed that the serum CysC levels were increased after acute MIS, and higher serum CysC levels at baseline might be an independent risk factor for PSCI in patients with acute MIS. Our study provides a new direction for the prevention of PSCI in MIS patients, but further clinical research are critical in examining whether reducing the level of serum CysC provides a potential preventive or therapeutic target for PSCI.

## CONFLICT OF INTEREST

The authors declare no conflicts of interest.

## AUTHOR CONTRIBUTIONS

*Conceptualization and design the study, interpretation of data, and drafting and revising the manuscript*: Xu Yan. *Data collection and analyses*: Huan Chen. *Critical revision of the manuscript for important intellectual content, study supervision, and fund support*: Xiu‐Li Shang. All authors have read and approved the final manuscript.

### PEER REVIEW

The peer review history for this article is available at https://publons.com/publon/10.1002/brb3.2519

## Data Availability

The data that support the findings of this study are available from the corresponding author upon reasonable request.
